# Absence of microsatellite instability in extramammary Paget's disease

**DOI:** 10.1002/ski2.37

**Published:** 2021-05-05

**Authors:** K. Kashiwada‐Nakamura, T. M. Myangat, I. Kajihara, Y. Kusaba, K. Tanaka, R. Sakamoto, S. Maeda‐Otsuka, S. Yamada‐Kanazawa, S. Sawamura, H. Kanemaru, Y. Nishimura, N. Honda, K. Makino, A. Miyashita, J. Aoi, T. Igata, T. Makino, S. Masuguchi, S. Fukushima, H. Ihn

**Affiliations:** ^1^ Department of Dermatology and Plastic Surgery Faculty of Life Sciences Kumamoto University Kumamoto Japan

## Abstract

**Background:**

Deficiency of DNA mismatch repair (MMR) induces microsatellite instability (MSI). Pembrolizumab, an antibody targeting PD‐1 (an immune checkpoint inhibitor), is more effective against MMR‐deficient tumours than against MMR‐proficient tumours. The status of MMR is a useful biomarker for predicting the effectiveness of pembrolizumab administration. Although the status of MMR has attracted attention in skin tumours, there are few reports on MSI in extramammary Paget's disease (EMPD).

**Objectives:**

To evaluate the status of MMR in patients with EMPD.

**Materials & Methods:**

One hundred one patients with EMPD were included. MMR status of the genomic DNA of each subject was analysed using Promega panel (approved as a companion diagnostic agent for the administration of pembrolizumab).

**Results:**

MSI testing showed the occurrence rates of MSI‐high (more than two markers are unstable), MSI‐low (one marker is unstable) and MSS (all markers are stable) tumour tissues were 0% (0/101), 1.0% (1/101) and 99.0% (100/101), respectively.

**Conclusion:**

The status of MMR may not be useful for the potential therapeutic application of pembrolizumab.

## Graphical abstract

To evaluate the status of DNA mismatch repair in patients with extramammary Paget’s disease (EMPD), 101 skin samples were analyzed using Promega panel (approved as a companion diagnostic agent for the administration of pembrolizumab). MSI testing showed the occurrence rates of MSI‐high (more than two markers are unstable), MSI‐low (one marker is unstable), and MSS (all markers are stable) tumor tissues were 0% (0/101), 1.0% (1/101), and 99.0% (100/101), respectively. The status of MMR may not be useful for the potential therapeutic application of pembrolizumab.

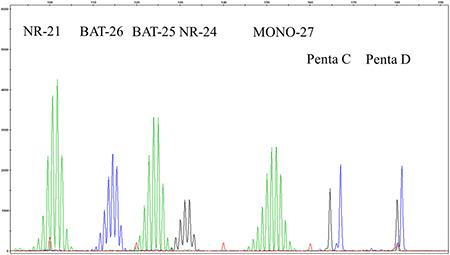

1

**What is already known about this topic?**

- Deficiency of DNA mismatch repair (MMR) induces microsatellite instability (MSI). Pembrolizumab, an antibody targeting PD‐1 (an immune checkpoint inhibitor), is more effective against MMR‐deficient tumours than against MMR‐proficient tumours. The status of MMR is a useful biomarker for predicting the effectiveness of pembrolizumab administration. Although the status of MMR has attracted attention in skin tumours, there are few reports on MSI in extramammary Paget's disease (EMPD).

**What does this study add?**

- MSI testing showed the occurrence rates of MSI‐high (more than two markers are unstable), MSI‐low (one marker is unstable) and MSS (all markers are stable) tumour tissues were 0% (0/101), 1.0% (1/101) and 99.0% (100/101), respectively. The status of MMR may not be useful for the potential therapeutic application of pembrolizumab.

## 2 INTRODUCTION

Deficiency of DNA mismatch repair (MMR) induces microsatellite instability (MSI).^1^ The number of somatic mutations in MMR‐deficient tumours is approximately 24.4 times more than that in MMR‐proficient tumours.^2^ Tumours with a high tumour mutational burden (TMB) are easily recognized by a patient's immune system because of the presence of more neoantigens compared to those in tumours with a low TMB.^3^ Pembrolizumab, an antibody targeting PD‐1 (an immune checkpoint inhibitor), is more effective against MMR‐deficient tumours than against MMR‐proficient tumours. Thus, the status of MMR is a useful biomarker for predicting the effectiveness of pembrolizumab administration.^4^

There are several reports about the status of MMR in skin tumours, although the reported frequency varies according to evaluation methods, histological types and patient cohorts. The frequency of MSI ranges from 35% to 60%, from 11% to 25% and from 0% to 5% in sebaceous tumour,^5^, ^6^ melanoma^7^, ^8^ and basal cell carcinoma (BCC),^9^, ^10^ respectively. In contrast, MSI was not detected in mycosis fungoides.^11^ There are only two reports on the status of MMR in extramammary Paget's disease (EMPD). In a study by Stasenko et al.,^12^ sequence analysis had revealed that all analysed tissue samples (*n* = 23) were microsatellite stable (MSS). Furthermore, in a study by Kang et al.,^13^ the Promega panel (approved as a companion diagnostic agent for the administration of pembrolizumab) for the detection of MSI revealed that the percentages of MSI‐high, MSI‐low and MSS tumour tissues in 20 Chinese patients were 5% (1/20), 30% (6/20) and 70% (14/20), respectively.

The aim of this study was to assess the status of MMR in EMPD because there are very few methods of treating advanced EMPD.^14^ Therefore, we used the Promega panel to evaluate the status of MMR, and hence MSI, in a large group of patients with EMPD, diagnosed at Kumamoto University.

## 3 MATERIALS AND METHODS

### 3.1 Subjects and specimens

The eligible subjects met the following criteria: a histological diagnosis of EMPD, and the availability of sufficient tissue in paraffin blocks for assessment using the MSI Analysis System version 1.2 (Promega). All patients with EMPD were diagnosed at Kumamoto University Hospital between January 2006 and March 2020. The clinical and demographic characteristics of the 101 subjects analysed in this study are as follows.

- Age: 74.7 ± 10.7 years (41 to 94 years).
- Sex: 49:52 (male: female).
- Degree of invasiveness: 82:2:17 (in situ: microinvasion: invasive).
- Lymph node metastasis: 84:16 (negative: positive).
- Organ metastasis: 93:7 (negative: positive).

One male subject was not assessed using image evaluation as per his will. Institutional review board approval and written informed consent for this study were obtained. The study and the procedures were conducted in accordance with the Declaration of Helsinki.

### 3.2 DNA isolation and analysis of MSI

Eight micrometre‐thick serial sections obtained from formalin‐fixed, paraffin‐embedded (FFPE) tissues were cut and attached to glass slides. For enriching tumour content, non‐tumour cells were removed by reference to haematoxylin‐and‐eosin stained slide. Genomic DNA was isolated using a QIAamp DNA FFPE Kit (Qiagen). MMR status of the genomic DNA of each subject was analysed using the MSI Analysis System version 1.2 (Promega) according to the manufacturer's protocol. Five mononucleotide markers (BAT‐25, BAT‐26, MONO‐27, NR‐21 and NR‐24) were used to determine the MMR status of each sample. Capillary electrophoresis was performed on the samples using an Applied Biosystems 3130 Genetic Analyser (Applied Biosystems).

## 4 RESULTS

**FIGURE 1:**
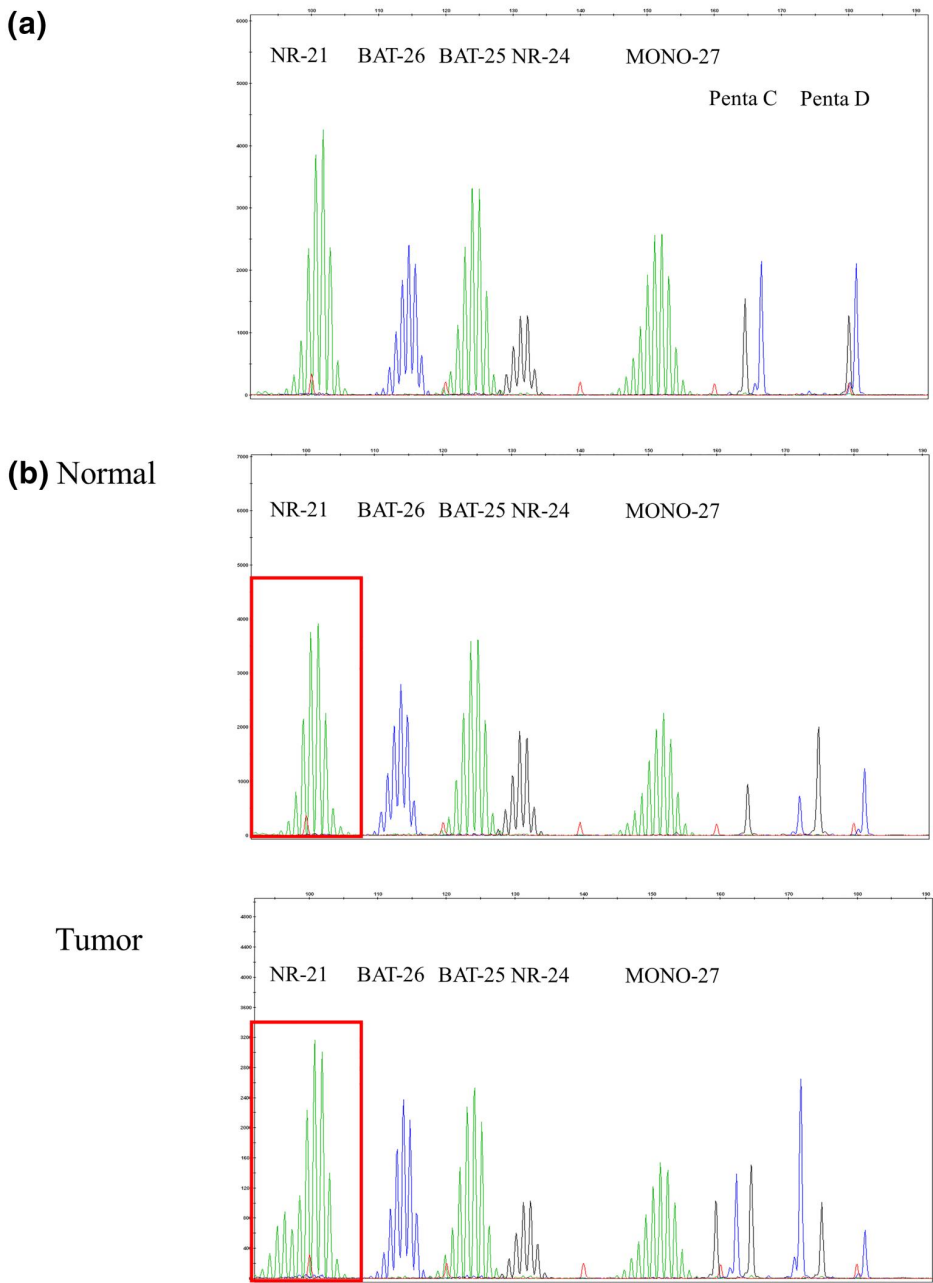
Results of capillary electrophoresis using the Promega Panel in extramammary Paget's disease (EMPD). (a) Representative result of microsatellite stable. (b) In one case of EMPD with an MSI‐low tumour, only the NR‐21 marker was present in the tumour tissue and was absent in normal tissue

None of the subjects in this study fulfilled the criteria for testing of Lynch syndrome.^15^ MSI testing showed that the occurrence rates of MSI‐high (more than two markers are unstable), MSI‐low (one marker is unstable) and MSS (all markers are stable) tumour tissues were 0% (0/101), 1.0% (1/101) and 99.0% (100/101), respectively (Figure 1a,b). In one case of EMPD with an MSI‐low tumour (83‐year‐old male, in situ invasiveness, no lymph node or organ metastasis), only the NR‐21 marker was present in the tumour tissue and was absent in normal tissue (consistent with MSI‐low) (Figure 1b).

## 5 DISCUSSION

The occurrence rate of MMR‐deficient tumours is about 2%–4% across multiple types of cancer except for uterine corpus endometrial carcinoma (30%) and colon adenocarcinoma (15%),^16^ and the assessment of MSI using Promega panel is presently the only approved biomarker for the administration of pembrolizumab in Japan. There are several reports about the status of MMR in skin tumours based on different analysis methods. As previously mentioned, in EMPD, the occurrence rates of MSI‐high, MSI‐low and MSS tumour tissues in 20 Chinese patients were 5%, 30% and 70%, respectively.^13^ Thus, to reveal the potential therapeutic application of pembrolizumab, we investigated the status of MSI using the Promega panel in a large group of patients with EMPD.

In this study, there were no MSI‐high tumour tissues in EMPD, although Kang et al.^13^ had reported that the occurrence rate of MSI‐high tumour tissue was 5%. Our results suggest that MSI may not contribute to the pathogenesis of EMPD. This discrepancy may depend on the differences among patient cohorts. Our study was based in a single institution, although it consisted of a large number of samples. Further investigations, including multicentre studies, are necessary to resolve this discrepancy.

In addition, pembrolizumab administration has recently been approved for unresectable and metastatic tumours with a high TMB (more than 10 mutations per megabase). Because the range of TMB in EMPD is reported to be from 0.06 to 13.5,^17^ our results suggest that the investigation of TMB, rather than that of MSI, may be suitable for the analysis of the possible application of pembrolizumab in EMPD.

In conclusion, although our study showed the absence of MSI in EMPD, the significance of MSI in other histological types of skin cancer needs to be analysed.

## CONFLICT OF INTERESTS

The authors declare that there are no conflict of interests.
